# Relation Between Renin–Angiotensin–Aldosterone System Inhibitors and COVID-19 Severity

**DOI:** 10.7759/cureus.22903

**Published:** 2022-03-06

**Authors:** Mousa J Alhaddad, Mohammed S Almulaify, Abdullah A Alshabib, Albatool A Alwesaibi, Mohammed A Alkhameys, Zainab K Alsenan, Hawra J Alsheef, Mohammed A Alsaghirat, Mohammed S Almomtan, Marai N Alshakhs

**Affiliations:** 1 Internal Medicine, Dammam Medical Complex, Dammam, SAU

**Keywords:** severe acute respiratory syndrome coronavirus 2 (sars-cov-2), angiotensin-converting enzyme inhibitors, hypertension, renin-angiotensin-aldosterone system (raas), angiotensin receptor blockers, coronavirus disease 2019 (covid-19)

## Abstract

Background: Angiotensin-converting enzyme 2 (ACE2) receptor serves as a receptor for severe acute respiratory syndrome coronavirus 2 (SARS-CoV-2), the virus causing COVID-19, to enter the lungs. Angiotensin-converting enzyme inhibitors (ACEIs) and angiotensin receptor blockers (ARBs) may increase the expression of ACE2, resulting in concerns that patients with COVID-19 who are receiving these agents may be at increased risk of severe disease. This study was conducted to further investigate the effects of ACEIs and ARBs on the severity of COVID-19 in hospitalized hypertensive patients.

Methods: The study was a retrospective observational study. The medical records of all adult hypertensive patients who were hospitalized at Dammam Medical Complex (DMC) between March 1, 2020, and December 31, 2020, due to COVID-19 were reviewed. The hypertensive patients who were receiving ACEIs or ARBs were compared with the other hypertensive patients who were not on ACEIs or ARBs.

Results: A total of 148 hypertensive patients were included in the analysis. They consisted of 106 male and 42 female patients (72% and 28%, respectively). Nearly half of the patients were Saudi (75 patients, 50%). A total of 81 patients were in the ACEI/ARB group, and 67 patients were in the control group. There were no differences between the two groups in age, diabetic status, history of chronic kidney disease, initial blood pressure measurements, and initial oxygen requirements, but the control group contained fewer female patients (18% versus 37%) and Saudi patients (36% versus 63%) than the ACEI/ARB group (p-values = 0.017 and 0.002, respectively). The use of ACEIs or ARBs was associated with significant reductions in ICU admission (9% versus 31%, p-value = 0.001), need for intubation (7% versus 28%, p-value = 0.002), and death (2% versus 24%, p-value = 0.000). A significant negative association between the use ACEIs or ARBs and mortality was also observed in the multivariate analysis after the adjustment for the possible confounders, with an odds ratio (OR) of 0.087 and a 95% confidence interval (CI) of 0.017-0.449.

Conclusions: ACEIs and ARBs have no adverse effects on the clinical prognosis of COVID-19 patients with hypertension. Their use might be even beneficial and protective, but future larger studies are needed to confirm these effects. In the meanwhile, they should be continued in COVID-19 hypertensive patients unless their use is contraindicated for other reasons (e.g., hypotension, hyperkalemia, or acute kidney injury (AKI)).

## Introduction

Severe acute respiratory syndrome coronavirus 2 (SARS-CoV-2), the virus responsible for COVID-19, was initially described in December 2019 in Wuhan, China [1]. The virus rapidly spread, and on March 11, 2020, the World Health Organization declared it a pandemic [2]. Researchers believe that the angiotensin-converting enzyme 2 (ACE2) receptor serves as a high-affinity receptor for SARS-CoV-2 to enter the lungs [3,4]. Angiotensin-converting enzyme inhibitors (ACEIs) and angiotensin receptor blockers (ARBs) may increase the expression of ACE2, resulting in concerns that patients with COVID-19 who are receiving these agents may be at increased risk of severe disease [5-7]. However, some recent studies concluded that ACEIs and ARBs do not modify the expression ACE2 and that they do not increase the replication of SARS-CoV-2 [8,9]. Moreover, contrary to the hypnotized negative effects of ACEIs and ARBs on COVID-19 patients, there are conflicting data that they might be beneficial. Their use was associated, for example, with lower serum levels of inflammatory markers, such as interleukin-6 (IL-6), C-reactive protein (CRP), and procalcitonin [10,11]. These drugs have been also reported to prevent hypokalemia, which is a common complication of COVID-19 [12]. Therefore, renin-angiotensin-aldosterone system (RAAS) inhibitors may lead to better outcomes by hindering the harmful effects of angiotensin II in systemic inflammation, as well as impeding the occurrence of hypokalemia.

Currently, the guidelines recommend that COVID-19 patients on ACEIs or ARBs should continue their use of these agents if there is no other reason for discontinuation (e.g., hypotension or acute kidney injury (AKI)) [13-15]. This study was conducted to further investigate the effects of ACEIs and ARBs on the severity of COVID-19 in hospitalized hypertensive patients.

## Materials and methods

The study was a retrospective observational study. The medical records of all adult hypertensive patients who were hospitalized at Dammam Medical Complex (DMC) between March 1, 2020, and December 31, 2020, due to COVID-19 were reviewed.

The inclusion criteria were polymerase chain reaction (PCR)-confirmed SARS-CoV-2 infection, age ≥ 18 years, and hypertension. The exclusion criteria included the patients who were managed in the outpatient settings and patients who were not on any antihypertensive medications at admission.

The hypertensive patients who were receiving ACEIs or ARBs (the ACEI or ARB group) were compared with the other hypertensive patients who were not on ACEIs or ARBs (the control group). The primary outcome was the mortality rate with the intubation and ICU admission rates as the secondary outcomes.

Data were analyzed using the Python programming language (version 3.7.6) with the use of the SciPy library (version 1.4.1) and Statsmodels module (version 0.11.1). Descriptive statistics (i.e., mean, standard deviation (SD), count, and percentage) were calculated as necessary. Categorical variables were compared using the Chi-square test, and continuous variables were compared using the two-sample t-test. A multivariate logistic regression model was created with death as the dependent variable. The model was adjusted for age, sex, nationality, diabetes, chronic kidney disease, use of ACEIs or ARBs, and oxygen requirements at admission. The odds ratios (ORs) and confidence intervals (CIs) were reported. A p-value of less than 0.05 was assumed to indicate statistical significance.

The research project was approved and monitored by the institutional review board of Saud Albabtain Cardiac Center (SBCC-IRB-D-2020-08), and all data were used only for research purposes.

## Results

A total of 148 hypertensive patients were included in the analysis. They consisted of 106 male and 42 female patients (72% and 28%, respectively) with a male-to-female ratio of 2.5. Nearly half of the patients were Saudi (75 patients, 50%). A total of 86 patients (58%) were diabetic. Twenty-four of them (16%) had a known history of chronic kidney disease. Twenty-seven patients (18.2%) were on ACEIs at admission, while 54 patients (36.5%) were on ARBs. A total of 67 patients (45.3%) were not on ACEIs or ARBs but were on other antihypertensive medications such as calcium channel blockers (CCBs) or thiazide diuretics (TDs). Most of the patients (89 patients, 60.1%) were on a single antihypertensive agent (e.g., an ACEI alone or a CCB alone). On the other hand, 59 patients (39.9%) used two or more antihypertensive medications (e.g., an ACEI/CCB combination or a CCB/TD combination). The means ± standard deviations for the patients' systolic blood pressure and diastolic blood pressure at their first presentation to the hospital were 138.9 ± 23.0 and 80.7 ± 15.0 mmHg, respectively. More than half of the patients (82 patients, 55.4%) were maintaining their oxygen saturation at higher than 93% with no need for any supplemental oxygen in the emergency department. Thirty-seven patients (25%) were connected to nasal cannulas. Eleven patients (7.4%) were put on a simple face mask, and 15 patients (10.1%) needed non-rebreather face masks. The remaining three patients (2.1%) required either intubation or noninvasive mechanical ventilation. Table 1 presents the patients' demographics and clinical conditions as they presented to the emergency department.

**Table 1:** Patients' Demographics and Clinical Presentations (n = 148)

Characteristics		n (%)
Gender	Male	106 (71.62%)
	Female	42 (28.38%)
Age (Mean ± SD)		57.36 ± 13.65
Nationality	Saudi	75 (50.68%)
	Non-Saudi	73 (49.32%)
Diabetes Miletus		86 (58.11%)
Chronic Kidney Disease		24 (16.22%)
Using ACEIs		27 (18.24%)
Using ARBs		54 (36.49%)
Using Other Antihypertensive Medications		113 (76.35%)
Number of Antihypertensive Medications	Single	89 (60.14%)
	Two or more	59 (39.86%)
Initial Systolic Blood Pressure in mmHg (Mean ± SD)		138.93 ± 23.03
Initial Diastolic Blood Pressure in mmHg (Mean ± SD)		80.72 ± 15.09
Initial Respiratory Rate (Mean ± SD)		23.67 ± 5.29
Initial Oxygen Needs	No Supplemental Oxygen	82 (55.41%)
	Nasal Cannula	37 (25%)
	Simple Face Mask	11 (7.43%)
	Non-rebreather Mask	15 (10.14%)
	Intubation	2 (1.35%)
	BiPAP	1 (0.68%)

During the hospital stay, 28 patients (18.9%) and 25 patients (16.9%) needed ICU admission and intubation, respectively. Eighteen patients (12.1%) died. The remaining 130 patients (87.9%) were successively discharged. The discharged patients stayed in the hospital on average for 11.5 ± 9.0 days. The mean ± standard deviation for the admission to death time in the group of patients who died was 11.7 ± 7.0 days.

A total of 81 patients were in the ACEI/ARB group, and 67 patients were in the control group. There were no differences between the two groups in age, diabetic status, history of chronic kidney disease, initial blood pressure measurements, and initial oxygen requirements, but the control group contained fewer female patients (18% versus 37%) and Saudi patients (36% versus 63%) than the ACEI/ARB group (p-values = 0.017 and 0.002, respectively). The use of ACEIs or ARBs was associated with significant reduction in the development of acute kidney injury (14% versus 39%, p-value = 0.001), ICU admission (9% versus 31%, p-value = 0.001), need for intubation (7% versus 28%, p-value = 0.002), and death (2% versus 24%, p-value = 0.000). No statistical significance was detected in the development of pulmonary embolism or the admission to discharge time (p-values = 0.182 and 0.356, respectively). Table 2 shows the differences between the patients on ACEIs or ARBs and the patients in the control group.

**Table 2:** Comparison Between Patients on ACEIs or ARBs and the Other Patients (n = 148) * Significant at a p-value of less than 0.05 ^ Minimal oxygen requirements were defined as the need for no more than nasal cannula.

Characteristics	ACEIs or ARBs (n = 81)	No ACEIs or ARBs (n = 67)	P-Value
Female (n (%))	30 (37.04%)	12 (17.91%)	0.017*
Age (Mean ± SD)	58.26 ± 12.52	56.28 ± 14.93	0.383
Saudi (n (%))	51 (62.96%)	24 (35.82%)	0.002*
Diabetes Miletus (n (%))	51 (62.96%)	35 (52.24%)	0.251
Chronic Kidney Disease (n (%))	7 (8.64%)	17 (25.37%)	0.012
Systolic Blood Pressure (Mean ± SD)	136.23 ± 19.94	142.18 ± 26.07	0.118
Diastolic Blood Pressure (Mean ± SD)	79.52 ± 11.84	82.16 ± 18.26	0.29
Respiratory Rate (Mean ± SD)	23.28 ± 4.33	24.14 ± 6.27	0.333
Minimal Oxygen Needs^ (n (%))	71 (87.65%)	48 (71.64%)	0.025
AKI (n (%))	11 (13.58%)	26 (38.81%)	0.001*
PE (n (%))	8 (9.88%)	2 (2.99%)	0.182
ICU Admission (n (%))	7 (8.64%)	21 (31.34%)	0.001*
Intubation (n (%))	6 (7.41%)	19 (28.36%)	0.002*
Death (n (%))	2 (2.47%)	16 (23.88%)	0.000*
Admission to Discharge Time (Mean ± SD)	10.94 ± 8.04	12.43 ± 10.28	0.356
Admission to Death Time (Mean ± SD)	10.0 ± 4.24	11.94 ± 7.33	0.724

The multivariate logistic regression model reinforced the statistically significant negative association between mortality and the use ACEIs or ARBs with an odds ratio (OR) of 0.087 and a 95% confidence interval (CI) of 0.017-0.449. The OR of death in patients who presented to the emergency department with minimal oxygen requirements was also significant at 0.284 (95% CI: 0.084-0.96). No statistically significant associations were observed between the outcome and the other included variables (age, gender, nationality, diabetes mellites, and chronic kidney disease). Table 3 shows the detailed results of the logistic regression model.

**Table 3:** Multivariate Logistic Regression of Mortality (n = 148) * Significant at a p-value of less than 0.05 ^ Minimal oxygen requirements were defined as the need for no more than nasal cannula.

Characteristics	P-Value	Odds Ratio	95% Confidence Interval
Age	0.724	1.008	0.965–1.053
Male Sex	0.212	3.181	0.517–19.559
Diabetes Milieus	0.433	0.618	0.185–2.063
Chronic Kidney Disease	0.643	0.695	0.149–3.233
Saudi Nationality	0.060	3.402	0.949–12.2
Minimal Initial Oxygen Requirement ^	0.043*	0.284	0.084–0.96
Use of ACEIs or ARBs	0.004*	0.087	0.017–0.449

## Discussion

One of the most common comorbidities among COVID-19 patients is hypertension, which is also the most important risk factor to delay the SARS-CoV-2 virus clearance [16]. Some researchers have proposed that the administration of hypertension medications such as ACEIs or ARBs to COVID-19 patients can aggravate the severity of COVID-19 or increase the chances of healthy patients encountering COVID-19 [7]. Therefore, they advocated for the discontinuation/cautious use of such drugs in patients with COVID-19. As a result, some physicians stopped the use of ACEI and ARB drugs in COVID-19 patients.

Hence, this study, which included 148 patients, investigated the use of two groups of antihypertensive medications, angiotensin-converting enzyme inhibitors (ACEIs) and angiotensin receptor blockers (ARBs), in the setting of COVID-19. It was observed that the hypertensive patients with COVID-19 who used ACEIs or ARBs had less critical outcomes (acute kidney injury, ICU admissions, and intubation) than the hypertensive patients who were not on ACEIs or ARBs. Moreover, among the 18 patients who died in our study, only two patients were using ACEIs and ARBs, representing a significantly lower risk of death in the ACEI/ARB group.

Our results are in the line with the results of multiple published studies that reported that the use of RAAS inhibitors is not associated with increased short-term mortality in COVID-19 patients [17,18]. Our results that suggested a protective effect of ACEIs and ARBs are also consistent with the results of a growing number of studies that associated their use in COVID-19 patients a lower risk of mortality [19-22]. Furthermore, the benefits of ACEIs and ARBs are not limited to the risk of mortality and the outcomes of severity such as ICU admission and use of ventilators. RAAS inhibitor use has been associated with a lower incidence of COVID-19 diagnosis and hospitalization [23,24]. Their use was also associated with increased seropositivity (formation of antibodies) and enhanced viral clearance [10,17]. However, they remain a significant risk factor for developing AKI, contrary to our results, and consequently, patients using them need careful monitoring of the renal functions [25,26].

Our study has several limitations that should be considered. The first is the relatively small sample size. The second limitation is the retrospective nature of the study, which led to the absence of some clinically relevant data such as the duration and degree of hypertension and the usage duration of the antihypertensive medications. In addition, we did not report the patients' cardiac diseases (e.g., heart failure and ischemic heart disease) as data regarding these diseases are usually stored at a separate cardiac center. These diseases correspond to significant comorbidities that are linked to poor COVID-19 outcomes. It is less likely, however, that our findings would have changed if these diseases were included in the analysis since they are more likely to present in the ACEI/ARB group rather than the control group. The fourth limitation is that the ACEI/ARB group contained more female and Saudi patients who might have been healthier from the start than the patients in the other group. Future randomized clinical trials are needed to overcome this issue. However, it seems that even after adjusting the effects of ACEIs and ARBs for the possible confounding variables, they remain to have a significant correlation toward better outcomes. Therefore, we would strongly encourage patients to continue with RAAS inhibitor pharmacotherapy during the COVID-19 pandemic.

## Conclusions

RAAS inhibitors such as ACEIs and ARBs have no adverse effects on the clinical prognosis of COVID-19 patients with hypertension. Their use might be even beneficial and protective, but future larger studies are needed to confirm these effects. In the meanwhile, they should be continued in COVID-19 hypertensive patients unless their use is contraindicated for other reasons (e.g., hypotension, hyperkalemia, or acute kidney injury).
